# A Disgrace to England

**Published:** 1897-03-27

**Authors:** 


The Hospital

A Journal of

The Medical Sciences and Hospital Administration.

Vol. XXI.—No. 548.] [March 27, 1897.

### A Disgrace to England.

The report which has just been issued by the Departmental Committee which was appointed last autumn to inquire into the prevalence of contagious disease in the British army in India, reveals a condition of affairs so terrible in itself, so frightful in its consequences on the future population of England, and so dangerous to all those dwellers in India who depend on the continued supremacy of the British troops for their safety and very life, that we do not hesitate again to call attention to the matter, although it is not long since the same subject occupied our columns.

The present is a time when sanitation is almost a fetish. For the suppression of fevers, for the prevention of tuberculosis, and for the stamping out of small-pox, almost all the rights of man are interfered with. The entering of a man's home, the compulsory disinfection of his premises, the removal of persons suffering from certain ailments, and their practically compulsory detention long after they are well enough to return to work, merely on the plea that they are dangerous to others, the interference with whole trades, the legal limitations imposed on those who engage in the sale of milk or of meat, including the destruction of whole carcases without a pennyworth of compensation to the butcher on the plea that the animal has been affected with tuberculosis, the compulsory inoculation of infants with vaccinia, and endless other things which we need not here mention, are submitted to by freeborn Englishmen in the name of sanitation and for the prevention of disease. Yet there are diseases which are of the foulest and most loathsome which, for some strange reason or want of reason, we refuse to do anything to check.

At an enormous cost we maintain in India an army of 71,000 English troops, all young fellows, all in good health at the time they are sent out, and from the day they arrive in India, all surrounded by every precaution imaginable against every disease—except that one group of diseases of which we speak.

What is the outcome of all our trouble? This; that while the health of the army in regard to those diseases against which we take precautions has been steadily improving since the mutiny, in regard to these diseases to prevent which we take no steps things are getting worse and worse, till now we have got to such a pass that out of our army of 71,000 we have a permanent sick list of 3,200 constantly in hospital from this cause, besides many others treated outside, and that among even the nominally healthy there is a vast amount of partial disablement and unfitness for any but routine duties. Judging by the number of men who were found to be unfit for service in the Chitral Field Force, it is estimated that if the whole army in India were required to take the field, 8,880 men would have to be put down as useless from this one cause.

To recognise the full disgrace of our inertness in the matter, we must recognise what this Committee tells us of the condition of affairs in other armies. Taking the mean for three successive years, here we have the number of admissions per annum per 1,000 of strength: Germany, 27·3; Russia, 43·0; France, 43·8; Great Britain (Home), 203·7; (India), 438·0!

It does not appear that the recent increase in these diseases is the result of any especial tendency to immorality among the troops which have recently been sent out. On the contrary, drunkenness and other crimes appear to be less prevalent than formerly. The root of the evil would seem to be the temptations cast in the way of young fellows, who find them hard to resist, and the complete absence of all attempt to exercise any control over those by whom these diseases are spread. The responsibility for this undoubtedly lies directly with the House of Commons. The Indian Government did endeavour to cope with the evil, and is both ready and anxious to do so now. Parliament, however, has declared in favour of free trade in vice, and this is the result.

The members of the committee visited Netley Hospital, and they give a shocking account of the mass of human wreckage that they there encountered—young fellows under 25 years of age, disfigured, crippled, unable to return to their homes, incapacitated from earning a living, and many of them evidently fated only for the grave.

But besides those who are utterly destroyed by these diseases, what about those who escape more lightly and return to civil life? Perhaps that is an even more sad history still, and its details are to be traced in our women's hospitals, in our workhouses, in our hospitals for sick children, and in hundreds of little graves all the country over, for these diseases are not only transmissible but hereditary.

Why do we do all this? Why, when we place everyone else under control in the name of sanitation, do we permit this public traffic in these particular diseases in and around cantonments over which we possess the most absolute control? Why do we permit the ruin of our youth and the endangering of our empire? and why do we, rather than interfere with the immoral trade of a few women in India, expose the innocent women of England and their unborn children to all the horrors of syphilitic infection? Why, indeed?

